# Case Report: A rare case of pulmonary actinomycosis and concomitant small cell lung cancer

**DOI:** 10.3389/fmed.2025.1673197

**Published:** 2025-10-09

**Authors:** Shilin Fang, Jianli Wang, Xiang Zhu, Xiaoyan Gai, Yongchang Sun

**Affiliations:** ^1^Department of Respiratory and Critical Care Medicine, Peking University Third Hospital, Beijing, China; ^2^Department of Pathology, School of Basic Medical Sciences, Peking University Third Hospital, Peking University Health Science Center, Beijing, China

**Keywords:** pulmonary actinomycosis, small cell lung cancer, pulmonary nodules, concomitant diseases, differential diagnosis

## Abstract

Pulmonary actinomycosis is a rare chronic granulomatous infection with non-specific clinical and imaging manifestations, often misdiagnosed as lung cancer. We present the case of an elderly male smoker with two pulmonary space-occupying lesions on the left and right sides, respectively, both showing hypermetabolism on positron emission tomography-computed tomography (PET/CT). The CT-guided lung biopsy of the left mass confirmed pulmonary actinomycosis. After treatment with penicillin for 1 month, the left lung mass got resolved significantly, while the right lung nodule increased during this period. Subsequently, the right nodule was diagnosed as small cell lung cancer via the endoscopic ultrasonography combined with LungPro navigation-guided transbronchoscopic lung biopsy and underwent thoracoscopic resection. The coexistence of pulmonary actinomycosis and lung cancer is extremely rare. This case underscores the importance of thorough diagnostic evaluation and careful differentiation between infectious and neoplastic pulmonary conditions to ensure appropriate management.

## Introduction

Actinomycosis is a rare chronic granulomatous disease caused by filamentous Gram-positive anaerobic bacteria in the family *Actinomyceataceae*, characterized by the formation of sinus tracts and the discharge of sulphur granules (1, 2). Actinobacteria, a typically conditioned opportunistic pathogen, can reside in the oral cavity, dental caries, tonsil crypts, and other areas of a healthy human body. Pulmonary actinomycosis accounts for approximately 15% of actinomycosis (3) and is often caused by inhaling secretions containing *Actinomycete* particles due to poor oral hygiene.

The clinical manifestations of pulmonary actinomycosis can mimic those of other infectious lung diseases or malignancies, posing significant diagnostic challenges. There have been some cases in which patients initially suspected of lung cancer were eventually diagnosed with pulmonary actinomycosis (4, 5). The diagnostic challenge becomes even greater when pulmonary actinomycosis coexists with lung cancer, as seen in some rare instances (6). The situation of small cell lung cancer combined with pulmonary actinomycosis is rarely reported. Early identification of pulmonary actinomycosis is essential for appropriate management. Herein, we describe a case diagnosed with concurrent pulmonary actinomycosis and small cell lung cancer, highlighting the diagnostic challenges and clinical implications.

## Case presentation

A 69-year-old male was admitted to our hospital on August 26, 2021, with a 3-month history of intermittent cough and a 1-month history of hemoptysis. He was a retired primary school teacher of Han ethnicity in rural region of China, with an average economic status, and had a medical history included hypertension, chronic gastritis, and a 20 pack-year smoking history. He denied having a family history of genetic diseases. Three months ago, the patient developed cough accompanied by a small amount of yellowish-white sputum, without fever, chills, dyspnea, or chest pain. Hemoptysis began 1 month ago, presenting as a small amount of blood in the sputum.

**Figure 1:**
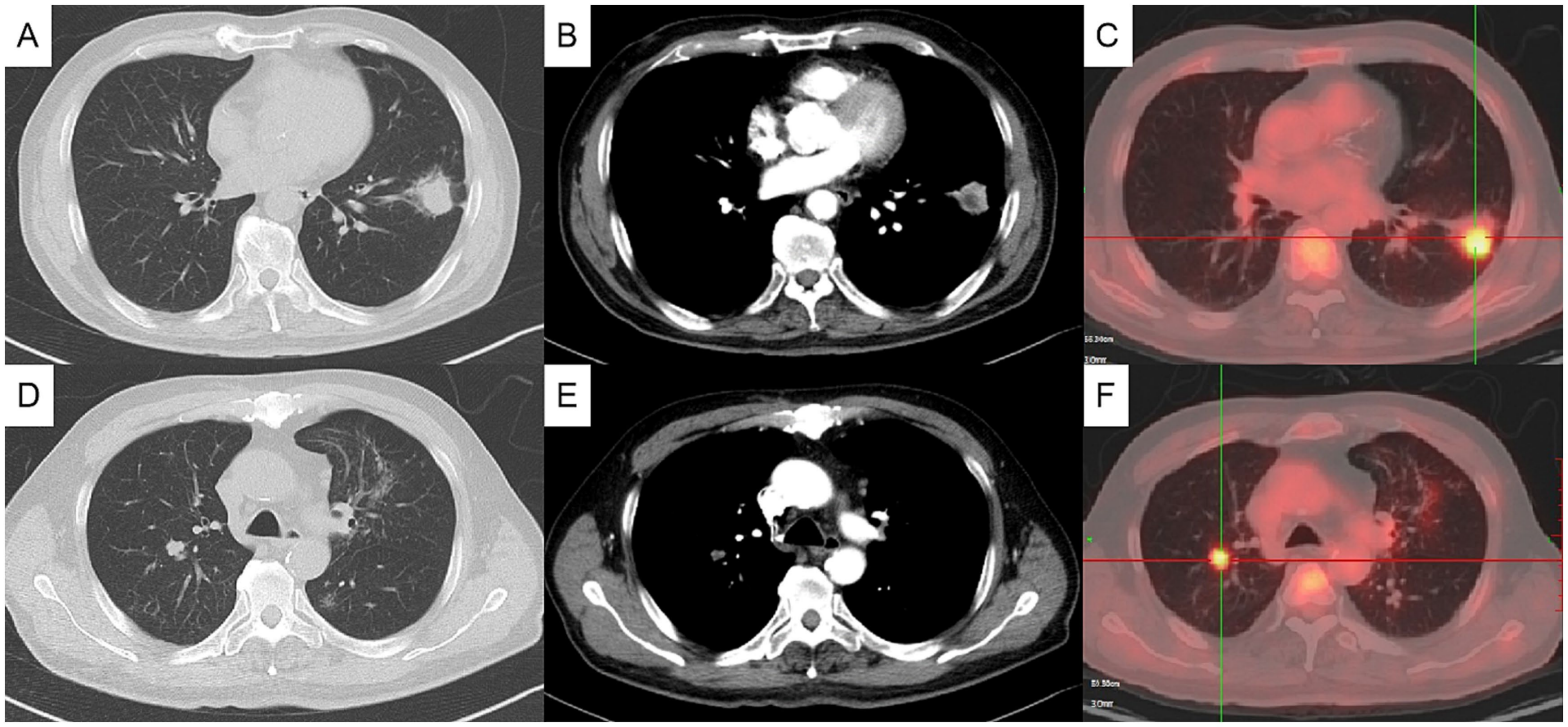
Initial chest computed tomography (CT) and positron emission tomography/computed tomography (PET/CT) (axial). Chest CT showing a mass in the anterior and inner basal segment of the left lower lobe, about 3.1×2.5 cm, with signs of lobulation, burr, pleural adhesion and local bronchial truncation **(A,B)**. Chest CT showed multiple small patch shadows around the bronchi of the right upper lobe and small solid nodule in the posterior segment of the right upper lobe, appropriate 1 cm in size **(D,E)**. PET/CT indicating hypermetabolism in the nodules of the left lower lung **(C)** and the right upper lung **(F)**, with SUVmax value of 5.7 and 4.7, respectively. (**A**, **C** pulmonary window, **B**, **D** mediastinal window).

**Figure 2:**
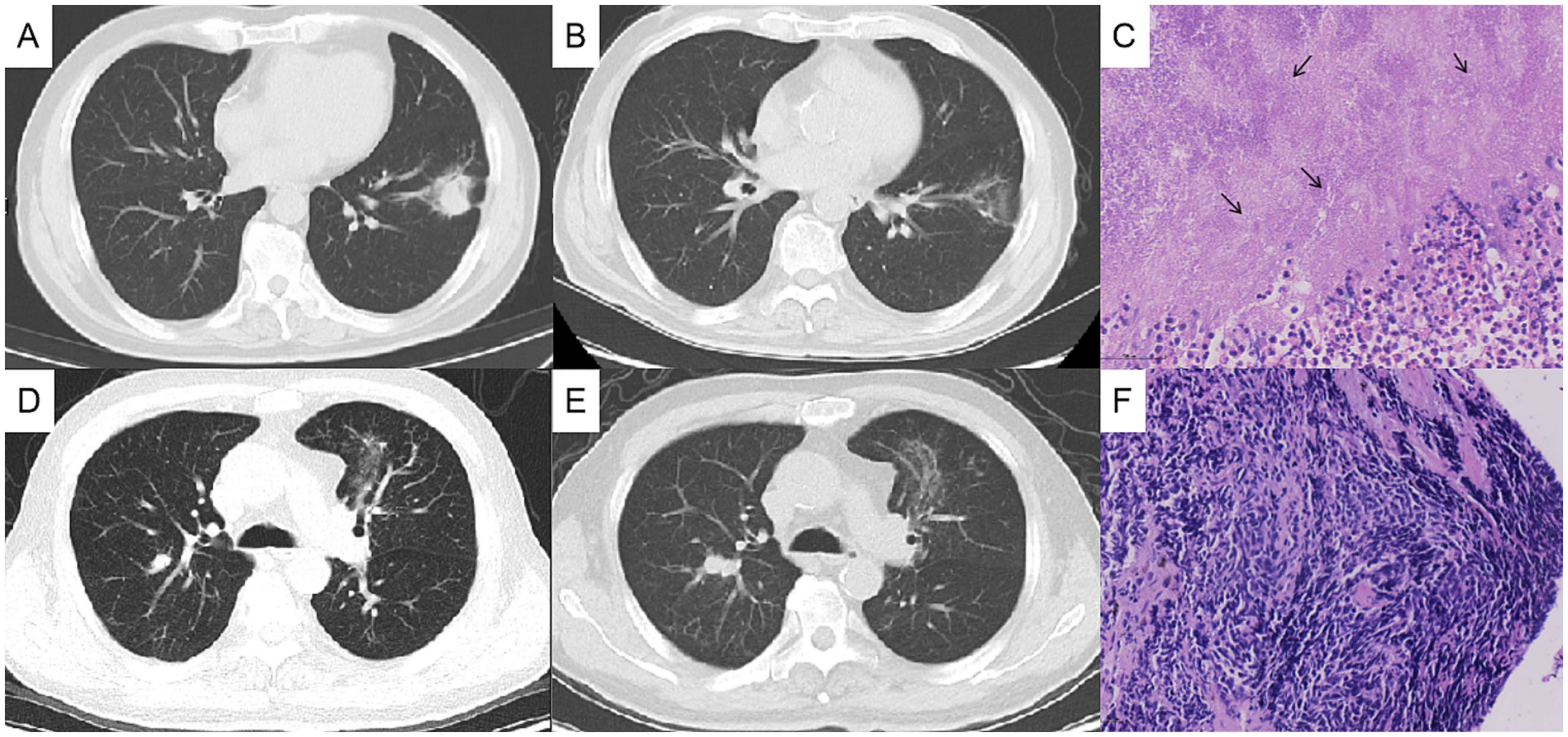
Chest computed tomography (CT) changes and pathological findings. The left lung mass, measuring approximately 3 cm before treatment **(A)**, showed significant reduction after 1 month of antibacterial therapy **(B)**, and the histopathological analysis of the lung biopsy confirmed actinomycosis **(C)**. The right lung nodule, initially about 1 cm in size before treatment **(D)**, increased to approximately 2 cm after 1 month of oral antibacterial therapy **(E)**, and the bronchoscopic biopsy pathologically confirmed the right pulmonary nodule as lung cancer **(F)**.

The physical examination showed no abnormality. The complete blood count, C-reactive protein (CRP), procalcitonin, erythrocyte sedimentation rate (ESR), G test, GM test, and tumor markers including CEA, SCC, CA125, pro-GRP and NSE were normal. T-SPOT was positive. Chest computed tomography (CT) depicted a 3-cm mass in the left lower lobe (Figures 1A,B) and a 1-cm nodule in the right upper lobe (Figures 1D,E). Positron emission tomography-computed tomography (PET/CT) indicated hypermetabolism in the left mass (SUVmax 5.7) (Figure 1C) and right nodule (SUVmax 4.7), both suggestive of malignancy (Figure 1F). The patient underwent a CT-guided percutaneous lung puncture biopsy of the left lower lobe mass. Pathological results revealed severe chronic active inflammation. Nodular amorphous necrotic substances were observed locally, containing thin rod-shaped thalli of varying lengths, some of which were clustered, indicative of actinomycosis (Figure 2C). Weak acid-fast staining of lung tissue was negative. Based on these findings, the patient was diagnosed with pulmonary actinomycosis. *β*-Lactams are recommended as first-line treatment of actinomycosis (7). Oral amoxicillin-clavulanate potassium (amoxicillin 0.2 g, clavulanate potassium 28.5 mg) were used empirically. The patient were prescribled 4 tablets orally twice daily, with a follow-up chest CT planned after 1 month. In addition, elevated blood glucose levels were detected during hospitalization, with glycosylated hemoglobin of 7.8%. He was diagnosed with type 2 diabetes, and started on hypoglycemic therapy.

After 1 month of antibacterial treatment, the patient’s cough and blood-streaked sputum were significantly reduced. A follow-up chest CT showed a marked reduction in the size of the left lung mass (Figures 2A,B). However, the right lung nodule was larger than before, with a diameter >2 cm (Figures 2D,E). He was readmitted to hospitalization for further evaluation of the right pulmonary nodule.

After admission, the tumor markers were retested, with a serum pro-GRP level of 100.1 ng/mL, significantly elevated compared to 60.5 ng/mL 1 month ago (reference range: 0–70 ng/mL). The patient underwent bronchoscopy under general anesthesia, which showed no abnormalities in bronchial lumen. The endoscopic ultrasonography combined with LungPro navigation-guided transbronchoscopic lung biopsy of the right lung nodule was performed, with the pathological diagnosis of small-cell lung cancer (Figure 2F), which was staged as cT1N0M0. The patient subsequently underwent a thoracoscopic lobectomy of the right upper lung in the thoracic surgery department.

The patient continued oral amoxicillin-clavulanate for 2 months postoperatively, after which follow-up CT confirmed complete resolution of the left lung actinomycosis and antibiotics were discontinued. Unfortunately, small-cell lung cancer recurred 1 year later, and despite chemotherapy, the patient developed multiple metastases and died 2 years later after surgery.

## Discussion

We presented a rare case of pulmonary actinomycetes coexisting with small cell lung cancer. The patient, an elderly male with diabetes and a 20 pack-year smoking history, exhibited space-occupying lesions in both lungs on chest CT. He was ultimately diagnosed with pulmonary actinomycosis in the left lung and small cell lung cancer in the right lung. The coexistence of pulmonary actinomycosis and lung cancer is extremely rare. This case presentation underscores the importance of thorough diagnostic evaluation and will be helpful for clinicians in improving their understanding of the diagnosis and differential diagnosis of pulmonary actinomycosis.

Pulmonary actinomycosis is a rare, chronic granulomatous infection that commonly occurs in men aged 11–20 years and 40–60 years (2). Risk factors are poor oropharyngeal hygiene, aspiration risk, underlying lung disease, immunosuppressive status, and prior actinomycotic infection (7). In this case, the patient had a history of heavy smoking and untreated diabetes, both of which are risk factors for pulmonary actinomycosis.

The clinical presentation of pulmonary actinomycosis is highly variable. The most common symptoms include cough, sputum production, and hemoptysis (5), with less frequent manifestations such as chest pain, dyspnea, fever, weight loss, and night sweats (3). Routine blood tests, such as white blood cell, ESR, and CRP, may be normal or mildly elevated, offering limited diagnostic value. Chest imaging varies, the most common chest CT findings are consolidation, sometimes presenting as mediastinal or hilar lymph node enlargement, atelectasis, cavitation, and ground-glass opacity, occasionally with pleural effusion (2, 5). These imaging features often overlap with those of other chronic infectious lung diseases, making differentiation challenging. Histopathologically, pulmonary actinomycosis typically demonstrates necrosis with yellow sulfur granules and filamentous Gram-positive fungus-like pathogens (7). In this case, pathology revealed inflammation and necrosis, with thin rod-shaped filamentous organisms of varying lengths. Both actinomycetes and Nocardia share filamentous and branched Gram-staining positive characteristics in histopathology, but Nocardia are weak acid-fast staining positive, which serves as a key distinguishing feature (8). For this patient, weak acid-fast staining is negative, which is more supportive of actinomycetes. In addition to traditional histopathology, metagenomics next-generation sequencing (mNGS) (9) and matrix-assisted laser desorption ionisation time-of-flight mass spectrometry (MALDI-TOFMS) (10, 11) are increasingly valuable for confirming actinomycosis.

Actinomycetes are usually extremely sensitive to *β*-Lactams, especially penicillin G and amoxicillin, which are considered the first-line treatment (7). Non-invasive infections are treated with oral antibiotics until 1 to 2 months after symptom resolution, while invasive infections, including severe suppuration, fistula/sinus, bone involvement, and massive hemoptysis, require high doses of intravenous antibiotics for 2 to 6 weeks, followed by oral therapy (2). In this case, amoxicillin-clavulanate potassium led to significant absorption of the left lung mass on CT scans.

Pulmonary actinomycosis are often “disguised” as lung cancer, and some patients are finally diagnosed only after surgical resection. Kim et al. summarized 94 cases of pulmonary actinomycosis, among which 33 (35.1%) patients were misdiagnosed as lung cancer (5). Another study summarized 36 histopathologically diagnosed cases, of which 10 patients underwent pulmonary lobectomy for final diagnosis (12). In recent years, many cases have been reported that the nodules on positron emission tomography/computed tomography (PET/CT) showed high uptake and were misdiagnosed as lung cancer and received unnecessary surgical resection (13, 14). In this case, biopsy of the left lung revealed thin rod-shaped filaments consistent with actinomycosis. After antibacterial treatment, the mass was absorbed, avoiding the surgical resection of the lower lobe of the left lung. This outcome was of substantial clinical significance for the patient and highlighted a critical diagnostic insight. Clinicians should maintain a high index of suspicion for lung cancer mimicking actinomycosis in cases of PET/CT-positive nodular lesions, and evaluate carefully to ensure accurate diagnosis and management prior to considering surgical management (15).

The coexistence of pulmonary actinomycosis and small cell lung cancer is exceedingly rare. We searched the literature about the coexistence of two diseases (6, 16). It has been reported that in patients with pulmonary nodules, the bacteria were cultured as pulmonary actinomycetes, but the nodules were not absorbed after antibacterial treatment. The pathology of surgical resection revealed concurrent actinomycosis and adenocarcinoma (6). In this case, the simultaneous presence of nodules in both lungs initially suggested a single etiology but was later confirmed to involve pulmonary actinomycosis and small cell lung cancer, highlighting the importance of considering multiple pathologies. This underscores the importance of considering multiple concurrent diseases when evaluating pulmonary nodules. Notably, risk factors such as smoking, diabetes, and immunosuppression increase susceptibility to both cancer and infection, predisposing patients to such coexistence (17–19).

## Strengths and limitations

The major strength of this report is the documentation of an exceptionally rare coexistence of pulmonary actinomycosis and small cell lung cancer, with detailed clinical, radiological, pathological, and therapeutic information. In addition, the use of navigation-guided bronchoscopy as a minimally invasive approach enabled accurate histological confirmation while reducing patient trauma, which may provide a useful reference for clinical practice. The main limitation is that this is a single case, and therefore the findings cannot be generalized or used to establish causal associations between infection and malignancy. Accumulation of additional cases in the future will be important to identify shared clinical patterns and to guide diagnostic and therapeutic strategies more robustly.

## Conclusion

Pulmonary nodule is common, and its differential diagnosis requires consideration of benign and malignant diseases. The diagnosis of pulmonary actinomycosis does not exclude the possibility of underlying lung cancer. In rare cases, pulmonary actinomycosis may coexist with pulmonary malignancy. This case emphasizes the need for comprehensive diagnostic evaluation and vigilance for multiple concurrent diseases, which is crucial for accurate diagnosis and optimal management.

## Data Availability

The original contributions presented in the study are included in the article/supplementary material, further inquiries can be directed to the corresponding author.
